# Hemoptysis Associated with Leptospirosis Acquired in Hawaii, USA

**DOI:** 10.3201/eid1712.110700

**Published:** 2011-12

**Authors:** Christopher A. Duplessis, Marvin J. Sklar, Ryan C. Maves, Anne Spichler, Braden Hale, Mark Johnson, Mary Bavaro, Joseph M. Vinetz

**Affiliations:** Naval Medical Center, San Diego, California, USA (C.A. Duplessis, M.J. Sklar, R.C. Maves, M. Johnson, M. Bavaro);; University of California School of Medicine, La Jolla, California, USA (A. Spichler, J.M. Vinetz);; Naval Health Research Center, San Diego (B. Hale)

**Keywords:** leptospirosis, hemoptysis, bacteria, Hawaii, severe pulmonary hemorrhagic syndrome, SPHS, zoonosis, travel

**To the Editor**: Severe pulmonary hemorrhagic syndrome (SPHS) is a serious complication of *Leptospira* infection, a globally widespread bacterial zoonosis that is increasing in incidence in tropical and subtropical regions. Despite decreasing endemicity of leptospirosis in industrialized regions, the disease is reemerging in travelers and recreationalists. Leptospirosis is an appreciable attributable cause of travel-related infections (typically associated with waterborne activities), and the incidence of travel-related leptospirosis is proportionally higher than that for endemic leptospirosis. Disease risk epidemiology has shifted concomitantly from occupational to recreational in industrialized countries (*1**–**3*). Risk factors include urbanization, climatic changes, and agricultural practices (*1**–**3*).

Clinical features of leptospirosis range from asymptomatic infections and undifferentiated febrile syndromes to multiorgan dysfunction and death. Weil syndrome (i.e., severe leptospirosis) is characterized by renal and hepatic dysfunction, hyperbilirubinemia (disproportionate to transaminase elevation), and hemorrhage (pulmonary, gastrointestinal, or intracranial). Pulmonary involvement predicts poor clinical outcome: the case-fatality rate for persons with SPHS is >50% (*4**–**6*). Most US leptospirosis cases are reported from Hawaii, where the annual incidence is 1.63 cases/100,000 person-years (*1*). Leptospirosis is endemic to Hawaii; however, SPHS is uncommonly reported (*7*).

We treated a 21-year-old active-duty Navy sailor for SPHS after he had a 5-day port visit in Hawaii, during which he went cliff-diving in Maunawili Falls. Afterwards, he returned to California and 2 days later sought medical attention in an outpatient clinic. Pharyngitis was diagnosed and azithromycin prescribed.

Two days later, he was hospitalized with fever, chills, pharyngitis, dyspnea, nonproductive cough, headache, myalgias, hemoptysis, epistaxis, diarrhea, nausea, emesis, meningismus, and a lower-extremity rash. Vital signs included temperature 38.3°C, pulse 132 beats/min, blood pressure 128/72 mm Hg, and oxygen saturation 98% on room air. Physical examination noted conjunctival suffusion, epistaxis, posterior cervical and inguinal lymphadenopathy, bilaterally diminished breath sounds, rhonchi and crackles, bloody cough, tachycardia, hepatosplenomegaly, and a macular rash over the lower extremities. Laboratory studies were noteworthy for reference range leukocyte count, hemoglobin (11.8 g/dL), platelets (102 × 10^3^/mm^3^), creatine phosphokinase (1,719 IU/L), sodium (128 mmol/L), bicarbonate (23 mmol/L), blood urea nitrogen (29 mg/dL), creatinine (2.2 mg/dL), aspartate aminotransferase (171 U/L), alanine aminotransferase (147 U/L), bilirubin (1.9 mg/dL), and urinalysis (7 erythrocytes and 9 leukocytes/high-power field). Chest radiography showed multilobar bilateral opacities, and cerebrospinal fluid (CSF) showed mild pleocytosis. The patient received intravenous acyclovir, ceftriaxone, and vancomycin and continued azithromycin.

At hospital admission, the patient experienced respiratory decompensation requiring endotracheal intubation and mechanical ventilation. Results of blood, urine, and CSF cultures and CFS PCR (herpes simplex virus and enterovirus) remained negative at 48 hours, prompting discontinuation of vancomycin and acyclovir. Serologic test results for HIV, dengue fever virus, mycoplasma, and *Chlamydophila* and *Rickettsia* species were negative. Nasopharyngeal influenza PCR, *Streptococcus pneumoniae* and *Legionella* spp. urinary antigen test results and hepatitis panel results were negative. *Leptospira* spp. test results by culture, PCR, and serologic testing (ELISA and microscopic agglutination testing) were negative.

Given an elevated suspicion for leptospirosis, ceftriaxone and azithromycin were continued through hospital day 7. The patient rapidly improved, was extubated after 48 hours, and was discharged on hospital day 7 with a 7-day course of oral doxycycline. A convalescent-phase serum sample had a titer of 1,600 against *L*. *interrogans* serovar Copenhageni, as determined by microscopic agglutination testing.

SPHS is associated with infection with *L*. *interrogans* serovars Copenhageni and Icterohaemorrhagiae (*8*), and the syndrome has been identified in diverse settings, including the Andaman Islands. Recent outbreaks have occurred in Nicaragua and Brazil (*4**,**5*). SPHS pathogenesis remains poorly understood. In animal models and human autopsy studies, immunoglobulin and complement are deposited along alveolar septa without a clear cause-and-effect relationship (*9*). Bacterial virulence factors are postulated but unproven. Leptospires induce endothelial activation and pulmonary endothelial and epithelial injury (possibly by immune-complex deposition and/or autoimmune mechanisms) (*9*). Pulmonary histopathology demonstrates a paucity of leptospires, and antigen levels do not correlate with injury severity (*9*). Steroids, intravenous immunoglobulin, and plasma exchange are of unproven benefit but have been reported to be useful (*9*). Genetically determined responses include associations with human leukocyte antigen–DQ6 and hyperactive Toll-like receptor 4–dependent immunity.

Diagnosis of leptospirosis may have been delayed for this patient because of early empiric azithromycin administration. Azithromycin is increasingly recognized as a potentially effective treatment that is comparable or superior to doxycycline (*10*) and thus warrants testing in human trials. Given the paucity of SPHS in leptospirosis case reports from Hawaii, potential sentinel cases may be harbingers of more virulent disease expression. A potential parallel is the emergence of SPHS in Salvador, Brazil, in 2003. No cases were identified before 2003, but 47 cases and a 75% case-fatality rate were identified during 2003–2005 (*4**,**5*). The entrenched active surveillance and physician awareness of SPHS in neighboring Brazilian cities suggests it is unlikely that this observation stemmed from prior underrecognition of disease; instead, it suggests de novo emergence.

Clinicians should consider leptospirosis (SPHS) in patients with acute fever accompanied by hemoptysis after travel to Hawaii, and leptospirosis should be suspected in any traveler with undifferentiated febrile illness, especially those reporting water exposures (*2*). Vigilant national surveillance is needed to determine further emergence of SPHS in Hawaii.
